# Microbe-driven immune suppression in colorectal cancer: the *Fusobacterium nucleatum* playbook

**DOI:** 10.3389/fimmu.2026.1795703

**Published:** 2026-03-25

**Authors:** Wang Wuyi, Pan Tao

**Affiliations:** Department of Colorectal Surgery, Sichuan Cancer Hospital and Institute, Sichuan Cancer Center, Cancer Hospital Affiliated to University of Electronic Science and Technology of China, Chengdu, China

**Keywords:** CD8+T cell exhaustion, colorectal cancer, *Fusobacterium nucleatum*, microbiome-targeted therapy, tumor immune microenvironment

## Abstract

Fusobacterium nucleatum, a key oral-pathobiont in colorectal cancer (CRC), has evolved from a presumed “passenger” to an active “co-conspirator” in tumorigenesis. This review systematically delineates its core role as an “architect of the immune microenvironment,” whereby it remodels T-cell immune responses through precise mechanisms to establish an immunosuppressive tumor microenvironment. Specifically, *F. nucleatum* employs virulence factors such as Fap2 and FadA for specific colonization, and achieves immune evasion by inhibiting NK and T-cell function and recruiting myeloid-derived suppressor cells (MDSCs). Post-colonization, it further fine-tunes T-cell subsets: driving Th17 polarization to create a pro-inflammatory milieu while suppressing the infiltration and function of CD8^+^ T cells and promoting their exhaustion. Intriguingly, it upregulates programmed death-ligand 1 (PD-L1) expression, which may conversely enhance tumor sensitivity to anti-PD-1/PD-L1 immunotherapy. Moreover, it collaborates with other microbes via quorum sensing and the oral-gut axis to construct a procarcinogenic ecosystem. Based on these mechanisms, *F. nucleatum* exhibits substantial clinical translational potential. Its load serves as an effective non-invasive diagnostic biomarker and a prognostic predictor, and may help predict responses to immune checkpoint inhibitors. Therapeutically, strategies targeting the eradication or inhibition of *F. nucleatum*—including antibiotics, specific phages, virulence factor inhibitors, and combination immunotherapy—represent highly promising novel directions. Despite existing challenges, future efforts to deepen mechanistic understanding, develop precision intervention tools, and establish integrated patient stratification systems hold the potential to revolutionize CRC prevention, diagnosis, and combination therapy by targeting *F. nucleatum*.

## 1 Introduction

In recent years, the rapid advancement of microbiome research has established the gut microbiota as a complex “microbial organ,” playing an increasingly prominent role in maintaining immune homeostasis and regulating disease progression (1). Particularly in colorectal cancer (CRC), the intricate interplay between the gut microbiome and the host immune system—especially T-cell immunity—constitutes a “microbiota-immune regulatory landscape” underlying tumor initiation, progression, and therapeutic response (2). This broad biological framework not only underscores dysbiosis as a key environmental etiological factor in CRC (3) but also provides a novel perspective for understanding the formation of the tumor immune microenvironment (TIME).

Among the trillions of gut microbes, Fusobacterium nucleatum—a common oral anaerobic opportunistic pathogen (4)—has emerged as a “star bacterium” in tumor microbiome research due to its significant and specific enrichment in CRC tissues and feces (5). It is no longer regarded as a mere “passenger” but as an active “colonizer” and “co-conspirator” in CRC pathogenesis (6). Substantial evidence indicates that *F. nucleatum* can directly intervene in host cell signaling, promote genetic and epigenetic alterations, reshape metabolic patterns, and profoundly influence the tumor immune microenvironment through various mechanisms (7). For instance, *F. nucleatum* promotes Th17 cell differentiation, inhibits CD3^+^ T-cell infiltration (8), and further modulates T-cell function and differentiation via metabolites such as short-chain fatty acids and formate (9), thereby fostering an immunosuppressive microenvironment conducive to tumor growth.

Why has Fusobacterium nucleatum become a focal point among the numerous CRC-associated microbes? Current research has identified several oncogenic or conditionally oncogenic bacteria linked to CRC development, including enterotoxigenic Bacteroides fragilis (ETBF), Escherichia coli carrying the pks genomic island, Parvimonas micra, and *Streptococcus gallolyticus (*10). However, compared to these microbes, *F. nucleatum* exhibits unique and irreplaceable biological characteristics across several key dimensions. Furthermore, studies have demonstrated that Fusobacterium nucleatum shows a significant enrichment trend in the early stages of colorectal cancer, particularly in high-grade adenomas and serrated adenomas (11). Its enrichment is associated with the CpG island methylator phenotype (CIMP) (12), suggesting it may promote the progression from adenoma to carcinoma through epigenetic regulatory mechanisms (13). Simultaneously, *F. nucleatum* can activate the Wnt/β-catenin signaling pathway via the FadA adhesin (14), inducing the secretion of pro-inflammatory cytokines such as IL-8 and CXCL1 (15), thereby creating a microenvironmental foundation conducive to epithelial cell proliferation and immune escape even in the initial stages of tumorigenesis.

First, regarding intratumoral colonization capability, *F. nucleatum* can achieve “precision homing” to tumor tissue via its adhesin Fap2, which specifically recognizes the Gal-GalNAc structure overexpressed on CRC cell surfaces—a feature not commonly observed in other CRC-associated bacteria (16). Second, in terms of immunomodulation, *F. nucleatum* is among the few bacteria demonstrated to directly interact with host inhibitory immune receptors via bacterial surface molecules, thereby directly suppressing NK cell and CD8^+^ T-cell function. In contrast, ETBF and pks^+^ E. coli primarily influence tumor immunity indirectly through toxin-mediated inflammatory responses or DNA damage (6).

More importantly, the enrichment of *F. nucleatum* in CRC is closely associated with upregulated immune checkpoint molecule expression (17), T-cell exhaustion phenotypes (8), and differential responses to immunotherapy (18), positioning it not only as a tumor-promoting factor but also as a potential modulator of immunotherapy response. Therefore, focusing on *F. nucleatum* as a research core facilitates a deeper understanding of the immune microenvironment remodeling in CRC within a highly integrated “microbiota–immunity–therapeutic response” framework.

This review aims to systematically elaborate on how *F. nucleatum* acts as a key “immune modulator,” reshaping T-cell immune responses through direct actions on immune cells or indirect alterations of the microenvironment, thereby potentially contributing to tumor progression, metastasis, and chemotherapy resistance in specific contexts (19). We will focus on dissecting its interaction networks with different T-cell subsets (CD8^+^ T cells, CD4^+^ T-cell subsets including Th17, Tregs, Tfh) and other immune cells (MDSCs, tumor-associated macrophages) (20, 21), and explore the considerable translational potential of targeting *F. nucleatum*—via probiotics, phages, metabolite modulation, and combination therapy with immune checkpoint inhibitors—in CRC diagnosis, prognosis, and treatment (22, 23). By concentrating on this specific pathogen, we hope to provide a clearer paradigm for understanding the microbial immunopathogenesis of CRC and pave the way for developing novel combination immunotherapies. Compared with prior reviews that broadly summarize Fusobacterium nucleatum in CRC, this review is organized around T-cell–centered immune remodeling and its relevance to immunotherapy responsiveness, integrating (i) intratumoral colonization/immune evasion modules, (ii) T-cell subset reprogramming (Th17/Treg/Tfh/CD8), and (iii) translational implications (biomarkers, interventions, and combination strategies). To improve rigor, we explicitly annotate levels of evidence throughout (clinical association *vs*. mechanistic *in vitro vs*. causal *in vivo vs*. pharmacologic reversibility) and highlight key confounders in microbiome-immune studies (diet, antibiotics, host genetics, tumor location, spatial architecture).

## 2 The immune “evidence” and “methods” of *Fusobacterium nucleatum*

### 2.1 Colonization and expansion: strategies for tumor tropism and immune evasion

Beyond its significant enrichment in advanced colorectal cancer (CRC) tissues, a growing body of longitudinal and cross-sectional studies confirms that *F. nucleatum* is already markedly enriched during the early stages of CRC development, including in high-grade adenomas and sessile serrated adenomas (SSAs) (11, 24–26). For instance, Ito et al. found that the abundance of *F. nucleatum* increases with the histological grade of colorectal tumors and is significantly associated with pre-neoplastic lesions exhibiting the CpG island methylator phenotype-high (CIMP-high), suggesting its potential role in promoting the adenoma-carcinoma sequence through epigenetic regulation (11). Mechanistically, *F. nucleatum* can activate the Wnt/β-catenin signaling pathway via its adhesin FadA (7) and induce the secretion of pro-inflammatory cytokines such as IL-8 and CXCL1 (15). This activity establishes a microenvironmental foundation conducive to epithelial cell proliferation and immune escape at the tumor initiation stage. This process does not rely on acute, robust inflammation but is characterized by persistent, low-grade immune remodeling. Consequently, available evidence supports the view that F. nucleatum may be more than a passive passenger in some CRC pathways and could contribute to early microenvironmental remodeling during the adenoma–carcinoma sequence; however, the extent of causality likely varies by host, tumor subtype, and microbial context (25, 26).

Successful colonization of CRC tissue by Fusobacterium nucleatum is a prerequisite for its pro-tumorigenic activity and relies on a combination of tumor-specific adhesion, epithelial barrier disruption, and active suppression of host immune surveillance. A central mechanism underlying its tumor tropism is the interaction between the bacterial outer membrane protein Fap2 and the D-galactose-β (1–3)-N-acetyl-D-galactosamine (Gal-GalNAc) moiety, a glycan structure aberrantly overexpressed on CRC cells (16). This highly selective molecular recognition enables *F. nucleatum* to preferentially anchor to and invade tumor tissue, thereby establishing a stable intratumoral niche. In parallel, the virulence factor FadA binds to E-cadherin on intestinal epithelial cells, triggering activation of the Wnt/β-catenin signaling pathway while simultaneously disrupting E-cadherin–mediated intercellular junctions (7). The resulting loss of epithelial barrier integrity not only facilitates bacterial infiltration but also permits increased entry of pro-inflammatory mediators into the tumor microenvironment.

**Figure 1:**
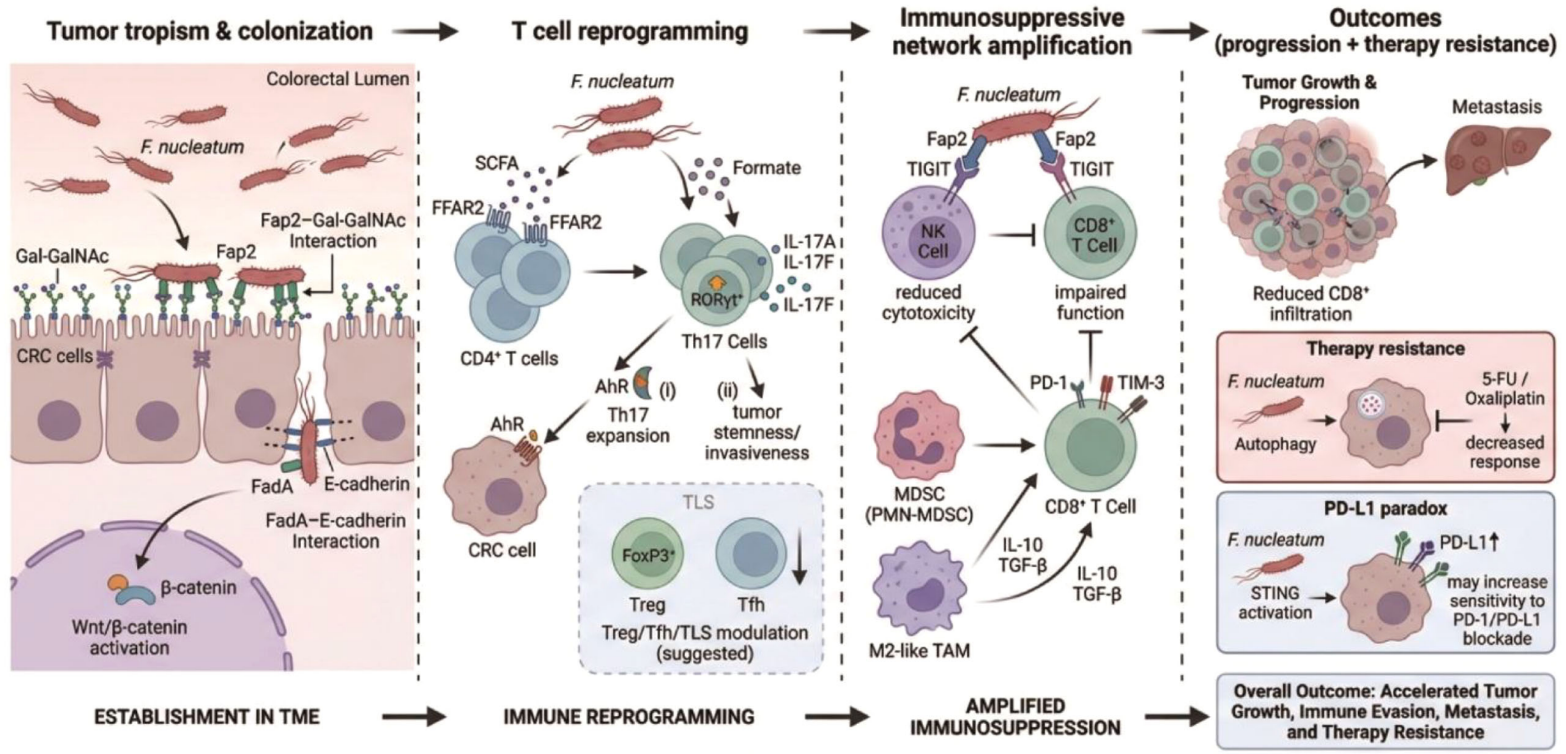
Fusobacterium nucleatum remodels T cell–centered immunity in CRC via adhesion, checkpoint hijacking, metabolites, and myeloid amplification. *F. nucleatum* preferentially colonizes CRC tissue via Fap2–Gal-GalNAc and FadA–E-cadherin interactions, disrupts epithelial junctions, and activates Wnt/β-catenin signaling. Bacterial metabolites (SCFAs–FFAR2; formate–AhR) promote Th17 programs, while Fap2–TIGIT signaling and myeloid suppressor circuits (PMN-MDSCs/M2-TAMs) inhibit NK/CD8^+^ T cell cytotoxicity and drive CD8^+^ T cell exclusion/exhaustion. The cumulative outcome is sustained immunosuppression with links to tumor progression and therapy resistance. Evidence note: Solid arrows indicate mechanisms supported by direct perturbation/causal experiments; dotted arrows indicate associative links, context-dependent effects, or hypotheses requiring prospective validation.

Beyond physical colonization, *F. nucleatum* actively circumvents immune surveillance during early stages of infection by engaging inhibitory immune checkpoints; specifically, Fap2 directly interacts with TIGIT expressed on natural killer (NK) cells and cytotoxic T lymphocytes, transmitting suppressive signals that attenuate cytotoxic function and cytokine production, thereby enabling immune evasion (27). Furthermore, *F. nucleatum* activates the nuclear factor-kappa B (NF-κB) signaling pathway, increasing the production of tumor-promoting cytokines such as IL-17A and TNF-α (28, 29), which further reinforces the immunosuppressive state within the tumor microenvironment. As colonization progresses, *F. nucleatum* further consolidates immune suppression by recruiting and activating myeloid-derived suppressor cells, particularly polymorphonuclear MDSCs. These cells secrete IL-6 and activate the calcineurin–NFAT signaling pathway, leading to downregulation of costimulatory molecules such as B7-H3 and B7-H4 on tumor cells, ultimately impairing CD8^+^ T cell–mediated antitumor immunity and establishing a sustained immunosuppressive tumor ecosystem (**Figure 1**).

Additionally, *F. nucleatum* can promote CRC metastasis by upregulating the expression of the long non-coding RNA KRT7-AS and its associated gene KRT7, a process also dependent on the activation of the NF-κB signaling pathway (30). *F. nucleatum* is not functionally uniform: strain-level variation in adhesins/virulence factors (Fap2, FadA) and tissue tropism likely contributes to heterogeneity in immune inhibition and clinical associations. Therefore, mechanistic conclusions drawn from selected isolates may not generalize across all CRC-associated *F. nucleatum* strains.

### 2.2 Manipulation and reprogramming: precision control of T cell immunity

Following stable colonization, Fusobacterium nucleatum emerges as a potent immunomodulator capable of precisely reprogramming T cell–mediated immunity within the colorectal cancer microenvironment (**Figure 1**).

A prominent feature of this regulation is its ability to promote Th17-driven inflammatory responses. In murine CRC models, intestinal colonization by *F. nucleatum* markedly increases the proportion of RORγt^+^ CD4^+^ T cells in the colonic lamina propria, accompanied by elevated expression of the effector cytokines IL-17A and IL-17F (31). This Th17 polarization is mediated through both direct and indirect mechanisms. Bacterial metabolic activity generates short-chain fatty acids (SCFAs) that engage free fatty acid receptor 2 (FFAR2) on CD4^+^ T cells, thereby directly facilitating Th17 expansion (31). In parallel, *F. nucleatum* stimulates intestinal epithelial cells to secrete chemokines such as IL-8 and CXCL1, which recruit neutrophils that cooperate with Th17 cells to establish a sustained pro-inflammatory network (15). Beyond classical inflammatory pathways, *F. nucleatum*–derived metabolites such as formate further amplify this effect by activating the aryl hydrocarbon receptor (AhR) signaling, enhancing tumor cell stemness while promoting Th17 cell expansion *in vivo (*32). Formate, as a gut-derived oncometabolite, plays a crucial role in *F. nucleatum*–driven tumor invasiveness (32). Through the convergence of these immune-intrinsic and microenvironmental mechanisms, *F. nucleatum* accelerates CRC progression (31, 32).

In addition to promoting Th17 polarization, *F. nucleatum* can influence other T cell subsets through various mechanisms. For instance, *F. nucleatum* infection can, via metabolites like SCFAs, activate the AhR pathway to promote the expansion of regulatory T cells (Tregs), thereby enhancing immune tolerance (20, 33). Furthermore, *F. nucleatum* can inhibit the function of follicular helper T cells (Tfh), impairing the formation of tertiary lymphoid structures (TLS) and consequently weakening the anti-tumor immune response (34). These findings indicate that *F. nucleatum* ‘s regulation of T cell immunity is a multi-layered, multi-target systemic process.

Concomitant with its pro-inflammatory effects, *F. nucleatum* profoundly suppresses antitumor cytotoxic immunity by impairing CD8^+^ T cell infiltration and function (**Figure 1**). Clinical analyses consistently demonstrate reduced infiltration of CD3^+^ T cells in *F. nucleatum*–positive CRC tissues, indicative of a globally restrained adaptive immune response (8, 18, 35). Clinical cohorts have reported an association between *F. nucleatum* enrichment/dysbiosis and transcriptional or phenotypic features consistent with impaired CD8^+^ T-cell effector function, including reduced cytotoxic programs and increased inhibitory receptor signatures. However, in most human CRC datasets these links remain correlative, and do not by themselves establish that *F. nucleatum* directly drives exhaustion rather than acting as a surrogate for broader inflammatory ecology, tumor subtype, or treatment exposure (18, 35). Recent work supports a hierarchy of CD8^+^ T-cell dysfunction (e.g., progenitor-exhausted *vs*. terminally exhausted states) with distinct implications for responsiveness to PD-1/PD-L1 blockade. Current evidence linking *F. nucleatum* to “exhaustion” does not yet resolve whether the bacterium preferentially shapes specific exhaustion trajectories or simply shifts the overall balance of dysfunctional states. Future studies combining spatial/single-cell profiling with controlled microbial perturbation will be required to map these trajectories. The immunosuppressive microenvironment shaped by *F. nucleatum* is not the result of a single mechanism but constitutes a complex systemic network involving various immune cell subsets, cytokines, metabolites, and surface receptors. This network is multi-layered and self-reinforcing (**Table 1**). Firstly, direct inhibition of NK and CD8^+^ T cell cytotoxic function is achieved through the binding of Fap2 to TIGIT (27). Secondly, the recruitment and polarization of myeloid-derived suppressor cells (MDSCs) and M2-type tumor-associated macrophages (TAMs) create an amplifying immunosuppressive loop through the secretion of inhibitory cytokines like IL-10 and TGF-β (29, 36, 37). Emerging evidence further suggests that *F. nucleatum* may exert indirect epigenetic effects on the tumor microenvironment. By downregulating the activity of methyltransferase-like 3 (METTL3) in tumor cells and reducing N6-methyladenosine (m6A) RNA methylation, *F. nucleatum* contributes to transcriptional programs associated with CRC metastasis, which may secondarily shape T cell dysfunction within the tumor niche (19).

**Table 1:** Mechanistic map: how Fusobacterium nucleatum reshapes T cell–centered immunity in CRC.

Module/stage	Key bacterial factor(s)	Host target/pathway	Immune/TME outcome (T cell–centered)
Tumor tropism & niche establishment	Fap2	Binds tumor-enriched Gal-GalNAc glycan on CRC cells	Preferential tumor anchoring → stable intratumoral niche
Barrier disruption + oncogenic priming	FadA	E-cadherin binding → Wnt/β-catenin activation; disrupts junctions	Promotes proliferation; barrier leakage facilitates inflammatory mediator influx
Pro-inflammatory recruitment circuitry	(Invasion-associated signaling)	Induces IL-8/CXCL1	Neutrophil recruitment; supports inflammatory network that secondarily drives T-cell dysfunction
Th17 polarization (immune reprogramming)	Microbial metabolites (SCFAs)	FFAR2 on CD4^+^ T cells → Th17 expansion	↑ RORγt^+^ Th17, ↑ IL-17A/IL-17F, pro-tumor inflammation
Formate–AhR axis (metabolite-driven)	Formate	Activates AhR signaling	Enhances cancer stemness/invasion and amplifies Th17 expansion *in vivo*
Direct immune checkpoint hijacking	Fap2	Binds TIGIT on NK cells/CTLs	Suppresses cytotoxicity/cytokines → immune evasion
Additional inhibitory receptor engagement	CbpF	Activates CEACAM1	Inhibits NK/T-cell function
Lymphocyte killing	Fap2, RadD	Pro-apoptotic effects on lymphocytes	Reduces effector lymphocytes → weaker antitumor immunity
Myeloid suppressor amplification	(Multiple cues)	Recruitment of MDSCs; M2 polarization via TLR4 → IL-6/STAT3/c-MYC and NF-κB/S100A9	Myeloid-dominant immunosuppression → impaired CD8^+^ T-cell infiltration/function
pMDSC calcineurin–NFAT loop (as written in your draft)	(Myeloid activation)	IL-6 → calcineurin–NFAT; ↓ B7-H3/B7-H4	Sustained suppression of CD8^+^ T-cell antitumor activity (costimulation dampening)
PD-L1 “Trojan horse” phenomenon	Fn-driven tumor signaling	STING → PD-L1 upregulation (organoid-supported in your text)	Paradox: immunosuppression + potential sensitization to PD-1/PD-L1 blockade
Broader T-cell subset skewing (as written in your draft)	Metabolites/ecosystem effects	AhR-linked Treg expansion; Tfh/TLS impairment	↑ tolerance (Tregs); weakened TLS-associated antitumor response

Notably, the immunological consequences of *F. nucleatum* are highly context dependent and extend beyond direct T cell modulation. Despite its overall suppressive impact on cytotoxic immunity, *F. nucleatum* has been shown to upregulate PD-L1 expression on tumor cells, introducing a paradoxical dimension to immune regulation (38). Mechanistically, preclinical and organoid systems suggest that *F. nucleatum*–linked inflammatory sensing pathways can upregulate PD-L1, raising a testable hypothesis that a subset of PD-L1-high/*F. nucleatum* -enriched tumors could be more amenable to PD-1/PD-L1 blockade. Nevertheless, current human evidence is largely retrospective and confounded by MSI status, baseline immune infiltration, and other determinants of ICI response; therefore, these observations should be interpreted as hypothesis-generating rather than predictive (38). Supporting this concept, patient-derived organoid models demonstrate enhanced responses to anti–PD-L1 therapy in tumors harboring high *F. nucleatum* loads, potentially mediated through activation of the STING signaling pathway (38, 39). Beyond immune checkpoints, *F. nucleatum* orchestrates broader immune reprogramming by interfering with dendritic cell maturation, promoting the expansion of myeloid-derived suppressor cells, and driving polarization of tumor-associated macrophages toward an M2-like phenotype. The establishment of this systemic immunosuppressive network implies that single-agent immunomodulatory strategies often have limited efficacy in tumors with high *F. nucleatum* burden, providing a rationale for developing combination therapies targeting the microbiota-immune axis (38).

Beyond indirectly influencing tumor progression through immune cells, *F. nucleatum* can also act directly on colorectal cancer cells, activating multiple intracellular signaling pathways related to proliferation, survival, metabolism, and invasion. One of the most well-defined mechanisms is the binding of FadA to E-cadherin on epithelial cells, which activates the Wnt/β-catenin pathway, leading to the upregulation of cell cycle proteins like cyclin D1 and promoting cell proliferation (7). Concurrently, the lipopolysaccharide (LPS) of *F. nucleatum* can activate β-catenin via the TLR4/PAK1 cascade (40) and induce the expression of miR-21 through the TLR4/MYD88/NF-κB axis (41). miR-21 further drives cell cycle progression by inhibiting RASA1 and enhancing RAS-MAPK signaling. In terms of metabolic reprogramming, *F. nucleatum* infection can upregulate histone H3K27 acetylation levels, enhancing the expression of key glycolytic genes ENO1 and ANGPTL4, thereby promoting the Warburg effect in tumor cells and granting them a growth advantage in the nutrient-competitive tumor microenvironment (9, 42). These direct “bacterium-tumor cell” interaction mechanisms endow *F. nucleatum* with the dual role of both an “immune modulator” and a “metabolic co-conspirator” in CRC progression.

### 2.3 Cooperation and communication: construction of a tumor-promoting ecosystem

The tumor-promoting effects of *F. nucleatum* are not achieved in isolation but instead arise from its integration into complex microbial and host communication networks that reinforce oncogenic immunity (43).

**Figure 2:**
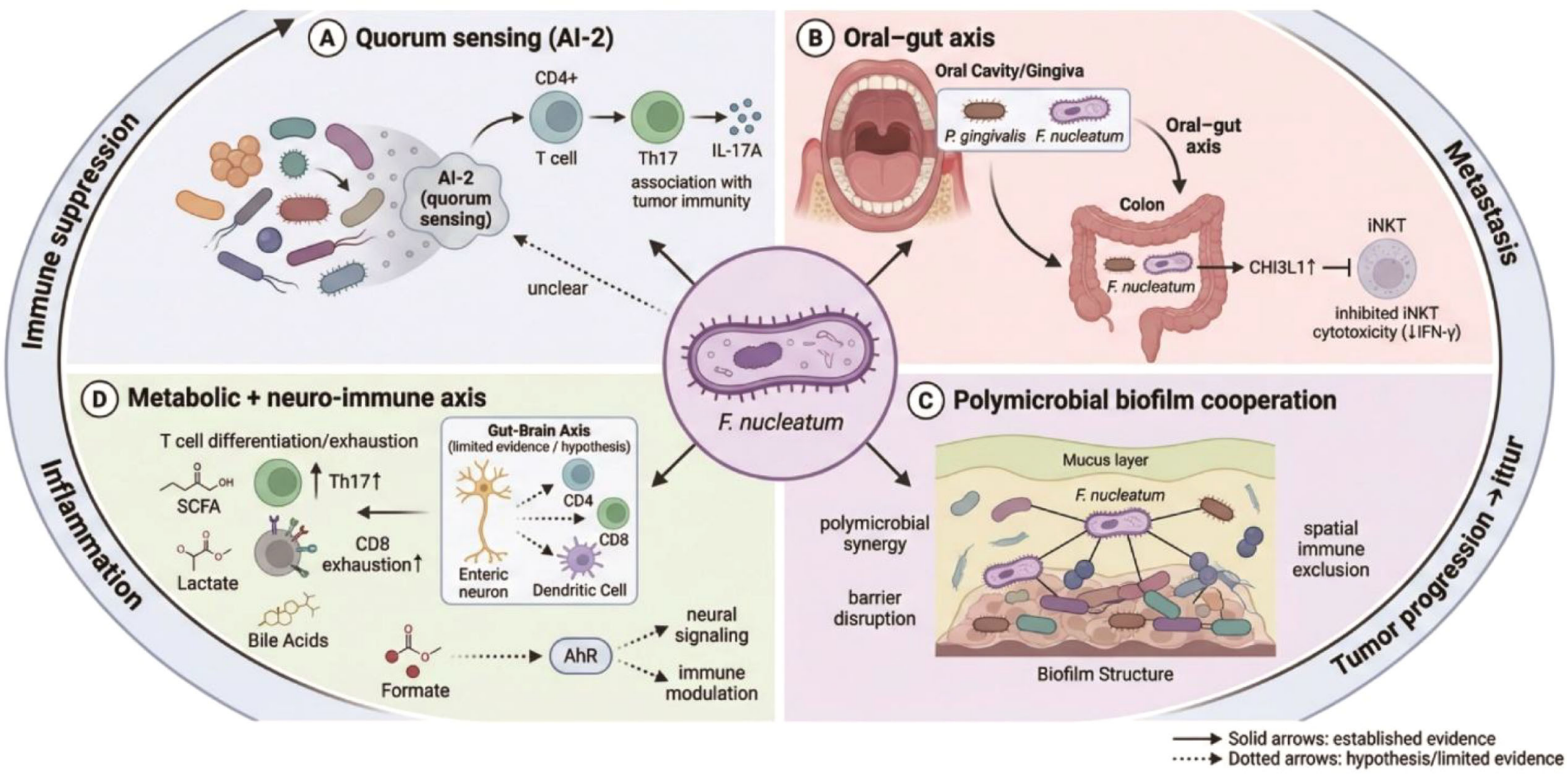
Fn-driven tumor-promoting ecosystem: quorum sensing, oral-gut axis, polymicrobial cooperation, metabolism, and neuro-immune crosstalk. Schematic illustration of *Fusobacterium nucleatum* (*F. nucleatum*) as an ecological hub that promotes colorectal cancer progression through multiple interconnected mechanisms. **(A)** Quorum sensing (AI-2): *F. nucleatum* participates in AI-2-associated interspecies communication linked to immune modulation, including CD4+ T-cell/Th17-associated responses. **(B)** Oral-gut axis: oral pathobionts, including *F. nucleatum*, may translocate from the oral cavity to the colon and contribute to metastatic and immunosuppressive niche formation. **(C)** Polymicrobial biofilm cooperation: *F. nucleatum* acts as a bridging organism in polymicrobial biofilms, promoting microbial synergy, barrier disruption, and spatial immune exclusion. **(D)** Metabolic and neuro-immune axis: *F. nucleatum*-associated metabolic remodeling and neuro-immune interactions may contribute to Th17 skewing, CD8+ T-cell exhaustion, and immune modulation. Solid arrows indicate established evidence; dotted arrows indicate hypothetical or limited-evidence pathways.

One such mechanism involves bacterial quorum sensing (QS), a density-dependent signaling system that enables coordination among microbial populations and is gaining attention for its role in immune regulation within CRC (43–46). Research has found significantly elevated levels of the universal QS signaling molecule autoinducer-2 (AI-2) in the feces, saliva, and tumor tissues of CRC patients, and these levels positively correlate with the abundance of CD3^+^ T cells in the tumor microenvironment (47). Although it remains unclear whether *F. nucleatum* directly produces AI-2, as a dominant species, it may indirectly influence T cell differentiation and function by modulating the overall QS network of the microbial community (43, 45, 47). For instance, AI-2 has been shown to be associated with Th17 cell differentiation, which may partly explain the enhanced Th17 response observed in *F. nucleatum* -enriched tumors (48). While the precise contribution of *F. nucleatum* to QS dynamics in CRC remains incompletely defined, these associations suggest that microbial interspecies “dialogue” plays a critical role in shaping the local T cell immune landscape (43, 45, 47) (**Figure 2**).

In addition to local microbial interactions, *F. nucleatum* functions as a central mediator of the oral–gut axis, linking oral dysbiosis to intestinal immune dysfunction (49–51). Periodontal pathogens such as Porphyromonas gingivalis, frequently co-detected with *F. nucleatum*, can alter gut microbial composition and promote intestinal immune dysregulation that synergizes with *F. nucleatum* to accelerate CRC progression (49, 50, 52). Mechanistically, P. gingivalis induces the expression of chitinase-3-like protein 1 (CHI3L1), which suppresses the cytotoxic activity of invariant natural killer T cells, thereby expanding the immunosuppressive network initially established by *F. nucleatum* (52). This inter-niche cooperation underscores the systemic nature of microbiota-driven immune modulation in CRC.

Beyond cellular and microbial communication, *F. nucleatum* also contributes to tumor progression through remodeling of the metabolic microenvironment (43, 53). Metabolic alterations within CRC tissues—including changes in short-chain fatty acids, lactate accumulation, and bile acid metabolism—are increasingly recognized as critical determinants of T cell fate and function (43, 53). Although *F. nucleatum* itself is not a canonical butyrate-producing bacterium, its enrichment can reshape microbial community structure and foster pro-inflammatory or immunosuppressive metabolic niches that indirectly govern T cell differentiation and exhaustion (43, 53). Elucidating how *F. nucleatum* interfaces with metabolic pathways will be essential for integrating tumor metabolism, microbiota dynamics, and immune regulation into a unified conceptual framework of colorectal carcinogenesis (43, 53).

Recent research has revealed that the gut microbiota can interact with the nervous system via the “gut-brain axis,” subsequently modulating immune function (32, 43). Metabolites produced by *F. nucleatum*, such as formate, may influence enteric neural signaling by activating receptors like AhR, thereby regulating T cell function (32, 43, 53). Furthermore, *F. nucleatum* infection may indirectly modulate the local immune milieu by altering intestinal barrier function and affecting the activity of gut neuroendocrine cells (32, 43). Although direct evidence linking *F. nucleatum* to neuro-immune regulation is currently limited, its potential role as a key node within the “microbiota-immune-neural” interactive network warrants further exploration (43).

### 2.4 Spatial ecology and metastatic seeding: biofilms, polymicrobial synergy, and immune conditioning beyond the primary tumor

An emerging concept in CRC microbiome research is that tumor-associated bacteria rarely act as solitary organisms; instead, they assemble into structured polymicrobial communities (biofilms) embedded within the mucus layer, thereby creating a stable platform for continuous immune modulation and oncogenic signaling. In this ecological framework, Fusobacterium nucleatum is often regarded as a “bridging” organism that promotes co-aggregation and spatial organization of diverse oral and gut taxa. Consequently, its tumor-promoting activity is frequently amplified at the community level, rather than reflecting a single-species effect alone (54).

In proximal/right-sided CRC, mucus-invasive biofilms are more frequently observed and have been linked to barrier disruption, chronic inflammation, and tumor progression. Such biofilm architecture can impose microenvironmental constraints—local hypoxia, nutrient competition, and metabolite gradients—that collectively impair immune cell positioning and sustained effector function, while enabling persistent myeloid-driven immunosuppression and inflammatory reinforcement (54).

In high-risk hereditary settings, including familial adenomatous polyposis (FAP), tumor-adjacent mucosa can harbor patchy colonic biofilms enriched for tumorigenic consortia, including enterotoxigenic Bacteroides fragilis (ETBF) and colibactin-producing Escherichia coli. These observations provide a mechanistic template for how genotoxicity and immunomodulation can converge within the same spatial niche: distinct community members contribute complementary functions (DNA damage, inflammatory amplification, and immune suppression), thereby generating a more stable pro-tumor ecosystem. Within such consortia, *F. nucleatum* may promote persistence and pathogenic output through its adhesive/co-aggregative capacity and immunoregulatory potential (55).

A spatial-ecology perspective may also help explain why *F. nucleatum* is more strongly associated with advanced stage, metastasis, and adverse prognosis in certain cohorts. On the one hand, *F. nucleatum* can disseminate with tumor-derived material and persist within metastatic lesions, suggesting that it is not merely a contaminant restricted to primary tumors (56). On the other hand, pan-cancer studies indicate that intratumoral bacteria are not uncommon and can display tumor-type-specific localization patterns (including intracellular residency), with the potential to reshape local immune states and therapy responses (57).

Polymicrobial communities and their metabolic outputs provide an additional mechanistic layer for immune shaping. Short-chain fatty acids (SCFAs), such as butyrate, can promote colonic regulatory T-cell (Treg) differentiation and function through epigenetic and metabolic mechanisms, thereby shifting the balance between inflammatory and suppressive T-cell programs and altering the amplitude of effector responses (58, 59). Consistent with this concept, defined commensal Clostridia species can induce colonic Tregs, further supporting a central role for the microbiota–Treg axis in mucosal immune homeostasis and tumor immune conditioning (60). Moreover, bile-acid-derived metabolites can directly influence Th17 versus Treg differentiation, implying that changes in microbial bile acid metabolism may systemically set the “immune tone” of T-cell responses (61, 62).

From a therapeutic standpoint, landmark studies demonstrate that gut microbial composition can influence the efficacy of immune checkpoint blockade (CTLA-4/PD-1/PD-L1) and modulate antitumor immunity through multiple mechanisms (63). In addition, the microbiota can shape the immune-mediated effects of chemotherapy—for example, the antitumor immune response elicited by cyclophosphamide is microbiota-dependent—highlighting the microbiome as an upstream determinant of chemo–immune coupling (64). Beyond systemic effects, intratumoral bacteria may even mediate drug resistance through direct metabolic inactivation of chemotherapeutics, underscoring intratumoral microbiota as a clinically relevant variable in treatment response and resistance (65).

Collectively, these spatial constraints can promote T-cell exclusion, limit antigen-presenting cell positioning, and sustain myeloid-dominant suppression—providing a plausible ecological route by which *F. nucleatum*–enriched communities shape T-cell dysfunction in CRC.

## 3 Clinical translation: from biomarkers to therapeutic targets

**Figure 3:**
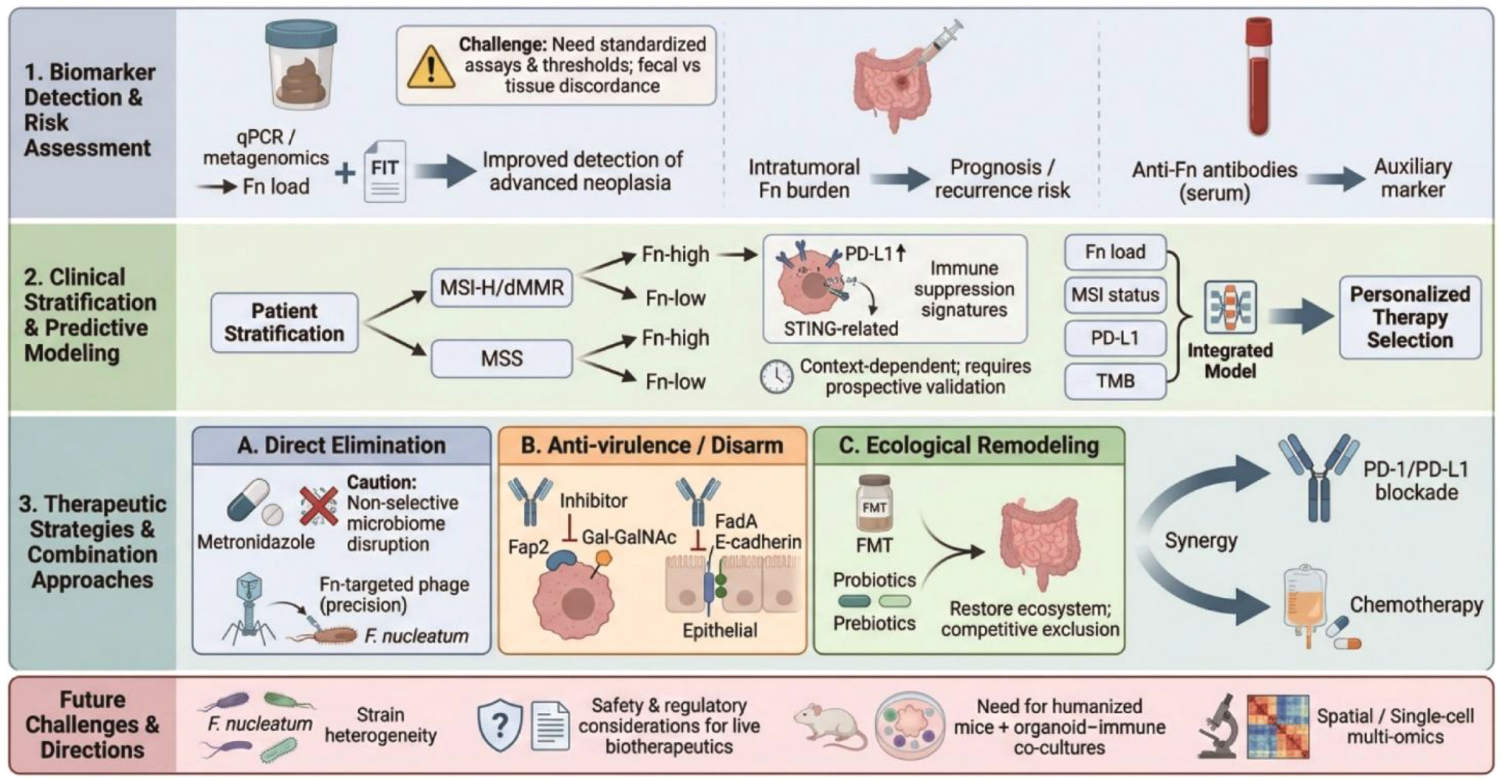
Clinical translation of Fn in CRC: from biomarkers to precision microbiome interventions and combination therapy. *F. nucleatum* can be quantified in stool and tissue for early detection and prognostic stratification, and may complement FIT or other molecular markers. Therapeutic concepts include depletion (antibiotics; Fn-selective phage approaches), anti-virulence blockade (Fap2/FadA), and ecological remodeling (FMT/probiotics), often envisioned in combination with chemo- or immunotherapy. Key barriers include assay standardization, strain heterogeneity, safety/regulatory constraints, and the need for prospective validation. Evidence note: Solid arrows indicate mechanisms supported by direct perturbation/causal experiments; dotted arrows indicate associative links, context-dependent effects, or hypotheses requiring prospective validation.

Associations between intratumoral/fecal *F. nucleatum* and immune phenotypes can be shaped by (i) diet and geography, (ii) antibiotic exposure and other medications, (iii) host genetics and immune set-points, (iv) tumor location (right *vs* left; mucinous features), and (v) spatial immune architecture (immune-excluded *vs* inflamed; biofilm proximity). These variables should be considered when interpreting causality, biomarker performance, and therapeutic generalizability. The central role of Fusobacterium nucleatum in colorectal cancer initiation and progression has rapidly propelled this organism from a subject of mechanistic investigation to a promising clinical biomarker and therapeutic target (8, 66) (**Figure 3**; **Table 2**).Accumulating evidence indicates that *F. nucleatum*–associated signatures can be exploited across multiple stages of CRC management, spanning early detection, prognostic stratification, and therapeutic intervention (67–69).

**Table 2:** Clinical translation: biomarkers and targeting strategies for *F. nucleatum* in CRC.

Domain	Readout/strategy	Sample/modality	Claimed utility (per your draft)	Key caveats to note
Diagnosis	Quantification of *F. nucleatum* DNA	Feces (qPCR/metagenomics)	Elevated in adenoma/CRC; noninvasive screening	Need assay standardization; cohort variability
Diagnosis (combo)	*F. nucleatum* + FIT	Feces	Improves sensitivity/specificity for advanced neoplasia *vs* FIT alone	Implementation requires validated cutoffs & reproducibility
Auxiliary diagnosis	Anti–*F. nucleatum* antibodies	Serum (IgA/IgG etc.)	Supportive biomarker	Potential confounding from other fusobacterial exposures
Prognosis	Intratumoral Fn burden	Tumor tissue	Associated with worse OS/DFS, recurrence	Tissue–feces discrepancy; positivity rates vary
Therapy resistance	Fn-linked chemoresistance	Tumor ecosystem	Promotes resistance via autophagy; may guide risk stratification	Still needs prospective validation for clinical decision-making
Beyond single taxa	Microbial classifier panels	Fecal metagenomic signatures	More robust cross-cohort discrimination than single organism	Requires harmonized pipelines and external validation
Direct depletion	Antibiotics (metronidazole)	Preclinical	Reduce intratumoral Fn, suppress tumor growth	Non-selective microbiome disruption; may impair therapy responses
Selective killing	Bacteriophage cocktails	Oral phage therapy (models)	Reduce Fn load; ↑ CD4^+^/CD8^+^ infiltration; enhance chemosensitivity	Translation, delivery, resistance, safety need trials
Anti-virulence	Block Fap2/FadA	mAbs/small molecules	Prevent adhesion/entry and downstream signaling without broad microbiome damage	Proof-of-concept stage; target validation needed
Microbiota + ICI	Fn targeting + PD-1/PD-L1 blockade	Combination regimens	Fn depletion remodels TME and may potentiate checkpoint blockade	Needs patient stratification; context-dependent effects
Ecological modulation	FMT/probiotics/prebiotics (*Roseburia intestinalis*)	Microbiome restoration	Competitive exclusion of Fn; rebalance systemic immunity	Safety/standardization; variable engraftment
Challenges	Strain heterogeneity; causal *vs* passenger; assay mismatch	—	Limits “universal” targeting & biomarker transferability	Needs longitudinal + strain-level functional studies

### 3.1 As a diagnostic and prognostic biomarker

As a diagnostic biomarker, *F. nucleatum* offers particular advantages due to its tumor-specific enrichment and detectability in non-invasive samples (67, 68). Quantitative detection of *F. nucleatum* DNA in fecal specimens has emerged as an effective screening approach, with metagenomic and targeted analyses consistently demonstrating significantly elevated bacterial loads in patients with colorectal adenomas and carcinomas compared with healthy individuals (67, 68). Importantly, multiple independent cohorts have validated its diagnostic performance (67, 68). Beyond stand-alone testing, integration of fecal *F. nucleatum* DNA quantification with fecal immunochemical testing (FIT) substantially improves sensitivity and specificity for advanced colorectal neoplasia, including CRC and high-grade adenomas (67). This combined strategy enables the identification of lesions that may be missed by FIT alone, offering a refined approach for population-level screening (67). In addition to fecal DNA testing, serum antibody levels against *F. nucleatum* can serve as an auxiliary diagnostic marker for CRC (69, 70). Furthermore, intestinal microbial metabolite profiles, such as alterations in bile acids and short-chain fatty acids, are closely linked to CRC development, and their synergistic effects with *F. nucleatum* may further enhance diagnostic sensitivity and specificity (68).

In the prognostic setting, a high intratumoral burden of *F. nucleatum* has been reproducibly associated with adverse clinical outcomes and is increasingly recognized as an independent predictor of poor prognosis (66). Elevated *F. nucleatum* levels correlate with reduced overall survival, shortened disease-free survival, increased postoperative recurrence, and resistance to standard chemotherapeutic regimens (71). These associations are strongly supported by large-scale clinical studies; for example, multicenter analyses involving hundreds of CRC patients have shown that approximately 10–15% of tumors are *F. nucleatum*–positive, and that positivity is significantly associated with inferior survival outcomes (8). Mechanistically, *F. nucleatum* promotes chemoresistance through activation of pro-survival pathways, including autophagy, thereby attenuating the cytotoxic effects of first-line agents such as 5-fluorouracil and oxaliplatin (72). Accordingly, assessment of tumor-associated *F. nucleatum* burden may aid in identifying high-risk patients who are more likely to exhibit treatment resistance and may benefit from intensified or alternative therapeutic strategies (66, 72).

Although *F. nucleatum* is among the most consistently reported CRC-associated bacteria, more clinically robust strategies typically move beyond single-organism readouts toward multi-taxon microbial marker panels with cross-cohort validation. Early work established the feasibility of using fecal microbiome profiles for CRC screening and for capturing microbial features associated with early lesions (73, 74). Subsequent studies further developed metagenomic feature–based classifiers, integrating *F. nucleatum* into broader diagnostic frameworks and improving discrimination across the adenoma–carcinoma continuum and across populations (75, 76).

Importantly, integrative cross-cohort efforts and meta-analyses have identified relatively consistent CRC-associated microbial “core signatures” and emphasized model generalizability and feature stability—an evidence level that is typically stronger than single-cohort correlations for clinical translation (77, 78). Moreover, combined metagenomic and metabolomic profiling has revealed stage-specific shifts in microbial composition and metabolic outputs during CRC progression, providing a basis for linking microbiome markers to disease stratification and prognostic assessment (79).

Given the strong effects of diet, geography, and antibiotic exposure on microbiome composition, reproducibility across cohorts and populations is essential. Multi-cohort analyses have identified CRC-associated microbial markers that recur across distinct populations and have proposed more “universal” marker sets, thereby offering a more rigorous statistical context for positioning *F. nucleatum* as a clinically actionable biomarker rather than a population-specific correlate (80, 81).

Reported abundance depends strongly on detection modality (targeted qPCR, 16S rRNA sequencing, shotgun metagenomics, or tissue-based staining/ISH) and on analytical pipelines (primer sets, reference databases, normalization, batch correction). Moreover, fecal measurements primarily reflect luminal shedding and community ecology, whereas intratumoral measurements capture tumor-resident niches and may correlate more directly with local immune architecture; discordance between stool and tissue readouts is therefore expected. For clinical translation, studies should report pre-analytic handling, cutoffs, and external validation, and—when feasible—pair stool and tissue profiling to define which compartment best predicts specific endpoints (screening *vs* prognosis *vs* therapy response).

### 3.2 Potential as a predictor and modulator of immunotherapy response

In CRC, MSI/dMMR status, TMB/neoantigen load, baseline CD8^+^ infiltration (“hot” *vs* “cold”), and spatial immune architecture are dominant predictors of checkpoint blockade benefit. Accordingly, any proposed role for *F. nucleatum* as a response modifier should be evaluated within subtype-stratified cohorts (MSI-H *vs* MSS; inflamed *vs* excluded phenotypes) and ideally tested in prospective designs with longitudinal sampling. With the increasing use of immune checkpoint inhibitors in mismatch repair-deficient/high microsatellite instability (dMMR/MSI-H) CRC, the potential of *F. nucleatum* as a biomarker for predicting immunotherapy response is gaining attention. Although F. nucleatum is typically linked to an immunosuppressive microenvironment, preclinical and organoid studies suggest that F. nucleatum–associated inflammatory signaling can coincide with PD-L1 upregulation on tumor cells, including via STING-related pathways (38). This observation supports a hypothesis-generating model in which PD-L1-high/F. nucleatum-enriched tumors might represent a biologically distinct subset worth testing for differential checkpoint blockade sensitivity. However, robust clinical evidence that F. nucleatum independently predicts immunotherapy benefit in CRC is currently lacking, and most available human data remain retrospective with substantial confounding by MSI/dMMR status, baseline immune infiltration, and spatial immune architecture. Therefore, the clinical value of F. nucleatum as a predictive biomarker should be assessed in prospective, subtype-stratified cohorts with longitudinal sampling, rather than inferred from limited retrospective trends (38). Patient-derived organoid findings and retrospective cohort signals should thus be interpreted as supportive but not definitive. Future work should prioritize standardized assays and harmonized cutoffs, and evaluate whether combining F. nucleatum metrics with established molecular/immune features improves prediction beyond MSI/dMMR and baseline immune contexture.

Supporting this hypothesis, patient-derived organoid models have demonstrated improved responses to anti–PD-L1 therapy in tumors with elevated *F. nucleatum* levels (38). Nevertheless, the predictive value of *F. nucleatum* across different CRC subtypes remains to be validated in prospective clinical trials (38).

Multiple independent clinical cohorts indicate that baseline gut microbiome composition and diversity correlate with clinical benefit from PD-1/PD-L1 blockade and align with features such as antigen presentation capacity, CD8^+^ T-cell infiltration, and inflammatory transcriptional programs within tumors (63, 82, 83). Crucially, these links extend beyond correlation: preclinical studies demonstrate that defined commensal communities can enhance antitumor immunity and improve checkpoint blockade efficacy, supporting a transferable causal component within the microbiota–ICB response axis (84–87).

Clinically, two studies in PD-1–refractory melanoma provided proof-of-principle that microbiome manipulation can partially overcome primary resistance: fecal microbiota transplantation (FMT) from responders, combined with anti–PD-1 therapy, induced objective responses in a subset of patients and promoted favorable immune remodeling within the tumor microenvironment (88, 89). Although conducted outside CRC, these trials establish a translational framework for designing “microbiome intervention + ICB” strategies in CRC and emphasize the need for prospective cohorts with longitudinal sampling to clarify whether *F. nucleatum* is a causal driver, a surrogate for broader dysbiosis, or both.

### 3.3 Targeting *F. nucleatum*: emerging therapeutic strategies and combination approaches

Given its established pathogenic role, *F. nucleatum* has emerged as a compelling direct therapeutic target. The exploration of strategies to neutralize its tumor-promoting effects spans multiple modalities, ranging from direct eradication to precise interference with its virulence mechanisms, often in rational combination with established therapies. Early preclinical investigations demonstrated that antibiotics active against anaerobes, such as metronidazole, can reduce intratumoral *F. nucleatum* burden and suppress tumor growth (56). However, the non-selective nature of broad-spectrum antibiotics poses a significant limitation, as they indiscriminately disrupt the commensal gut microbiota, potentially eliminating beneficial antitumor bacteria and compromising the efficacy of chemotherapy or immunotherapy (90). This drawback has fueled the search for more precise interventions. Bacteriophage therapy represents a highly specific alternative, leveraging viruses that selectively infect and lyse bacterial targets. Orally administered phage cocktails designed against *F. nucleatum* have shown promise in animal models, markedly reducing bacterial loads, remodeling the immunosuppressive tumor microenvironment—evidenced by increased infiltration of CD4^+^ and CD8^+^ T cells—and enhancing chemosensitivity, all while minimizing collateral damage to the broader microbial community (22) (**Figure 3**; **Table 2**).

A complementary strategy focuses on disarming the bacterium rather than eliminating it. Anti-virulence approaches aim to inhibit key bacterial factors essential for colonization and pathogenicity. This includes developing small-molecule inhibitors or monoclonal antibodies that block the function of critical adhesins like Fap2 and FadA, thereby preventing *F. nucleatum* from adhering to host cells and initiating its deleterious signaling cascades (7). Such strategies offer the potential to neutralize the bacterium’s tumor-promoting effects without exerting strong selective pressure for antibiotic resistance.

The profound immunomodulatory influence of *F. nucleatum* provides a strong rationale for combining these targeting strategies with immunotherapy. Preclinical evidence supports this synergy, showing that depletion of *F. nucleatum* can reshape the tumor immune landscape and potentiate the efficacy of PD-1/PD-L1 checkpoint blockade (38). This forms the basis for innovative “microbiota modulation + immune checkpoint inhibitor” regimens. Beyond direct bacterial clearance, other combination avenues are being explored. These include modulating immunosuppressive metabolites produced by *F. nucleatum* to restore CD8^+^ T cell function, potentially enhancing adoptive T cell therapies like CAR-T or TIL therapy. Furthermore, co-administration of immunostimulatory probiotics with *F. nucleatum* -targeting measures may synergistically remodel the tumor microenvironment toward a more anti-tumor state.

Finally, broader ecological modulation of the gut microbiome offers an indirect yet powerful approach to suppress *F. nucleatum*. Strategies such as fecal microbiota transplantation (FMT) or supplementation with defined probiotics (the butyrate-producer Roseburia intestinalis) and prebiotics aim to restore a healthy microbial equilibrium, leveraging interbacterial competition to antagonize pathogenic colonization by *F. nucleatum* and promote a systemic immune balance conducive to antitumor immunity (91). Collectively, these diverse and evolving strategies highlight *F. nucleatum* not only as a biomarker but as a dynamic therapeutic node, opening new avenues for precision microbiome intervention in colorectal cancer management.

Broad-spectrum antibiotics can cause collateral depletion of beneficial commensals and may unpredictably alter responses to chemo- or immunotherapy; phage approaches offer higher specificity but face challenges in delivery, resistance, manufacturing/quality control, and safety monitoring; ecological remodeling (FMT/probiotics/prebiotics) is conceptually attractive but requires standardized donor/strain selection, engraftment tracking, and long-term safety evaluation. Across modalities, patient stratification (tumor subtype, immune phenotype, and microbial context) will likely be essential to avoid inconsistent clinical outcomes.

### 3.4 Future perspectives and challenges

Despite these advances, significant challenges remain. Substantial strain-level heterogeneity exists among *F. nucleatum* isolates, complicating its use as a universal target (91). The causal versus passenger role of *F. nucleatum* demands further longitudinal studies (91). Practical limitations include the risk of unintended consequences from microbiome interventions, discrepancies between fecal and tissue-based detection, and a lack of standardized assays (68, 91).

Future research should leverage spatial transcriptomics, single-cell sequencing, and organoid-microbe co-culture systems to elucidate the spatiotemporal dynamics and functional heterogeneity of *F. nucleatum* within the TME. Concurrently, efforts to standardize detection methods, conduct prospective clinical trials, and develop safe and effective microbiota-targeting agents will be crucial for laying the foundation for “microecological precision therapy” in colorectal cancer.

## 4 Summary and perspectives

Fusobacterium nucleatum has emerged as a recurrently enriched member of the colorectal cancer (CRC) ecosystem and is increasingly linked to immune remodeling in the tumor microenvironment (8, 20). Accumulating evidence suggests that, in a subset of CRC contexts, *F. nucleatum* may contribute to shaping T cell–centered immune regulation through multiple molecular interactions and microecological effects (8, 20, 27). Across studies, *F. nucleatum* positivity is associated with features including immune-evasive colonization, reduced cytotoxic T-cell infiltration and/or effector programs, altered CD4^+^ T-cell polarization (e.g., Th17/Treg skewing), and reinforcement of myeloid-dominant immunosuppressive circuits; however, the strength and direction of these associations appear to be context dependent and are influenced by host, tumor, and treatment variables (8, 17, 20, 27, 31). Therefore, *F. nucleatum* may serve as a useful biomarker and a plausible therapeutic node, but its clinical utility will likely require assay standardization, subtype-stratified evaluation (e.g., MSI status and baseline immune infiltration), and prospective validation before it can be positioned as an independent driver of immunotherapy responsiveness (22, 67, 92).

Despite its promising potential as both a biomarker and a therapeutic target, the clinical translation of *F. nucleatum* -centric strategies faces several key challenges. Significant individual and tumor heterogeneity—including variations in gut microbiota composition, diet, and the tumor immune microenvironment among patients—affects its pathogenic role and treatment response (93). The lack of standardized detection methods, such as qPCR, metagenomics, or fecal DNA analysis, compromises the reliability and comparability of results (67, 94). Furthermore, substantial functional differences exist among *F. nucleatum* strains regarding virulence factor expression and immunomodulatory capacity, necessitating strain-level functional studies (94). Finally, safety concerns persist; interventions like broad-spectrum antibiotics or phage therapy may disrupt beneficial commensal bacteria, potentially affecting immunotherapy efficacy, highlighting the need for more precise therapeutic tools (22, 95).

Looking forward, several priorities will define progress in this field. Future research should integrate multi-omics data with spatial transcriptomics, single-cell sequencing, and patient-derived organoid models to elucidate the spatiotemporal dynamics of *F. nucleatum* within the tumor microenvironment and its interaction networks with the host immune system (96, 97). Parallel efforts must focus on establishing standardized detection methods, conducting prospective clinical trials, and developing safe and effective microbiota-targeting interventions to pave the way for “microecological precision therapy” in CRC (67, 92, 94).

Mechanistic investigations must move beyond species-level associations toward strain- and subtype-specific analyses to resolve heterogeneity in immune modulation and virulence factor repertoires (94). Interaction networks should be expanded to incorporate neural, metabolic, and inter-microbial signaling axes, including quorum sensing and cross-site microbial communication along the oral–gut axis (32, 47, 98). Establishing definitive causality will require advanced experimental systems that more faithfully recapitulate human immunity, such as humanized mouse models and patient-derived organoid–immune co-culture platforms, enabling precise microbial manipulation (92, 96). Ultimately, clinical translation will depend on integrated patient stratification frameworks that combine *F. nucleatum* burden, tumor immune features, and host genetic context to guide personalized interventions (8, 67, 92). Parallel advances in synthetic biology, phage engineering, and multi-omics–driven biomarker discovery, together with robust regulatory pathways for live biotherapeutics, will be essential to transform microbiome-based insights into safe and effective therapies for patients with CRC (22).
